# The Interventional Effects and Mechanisms of Lonidamine in Combination with Apigenin on Colorectal Cancer

**DOI:** 10.3390/cimb47100825

**Published:** 2025-10-08

**Authors:** Yi Zhou, Jiahao Shi, Mengjie Zhang, Hua Yang, Jian Fei

**Affiliations:** 1 School of Life Sciences and Technology, Tongji University, Shanghai 200092, China; 2011430@tongji.edu.cn (Y.Z.); 07015@tongji.edu.cn (J.S.); 98061@tongji.edu.cn (M.Z.); yanghua0712@126.com (H.Y.); 2 Shanghai Engineering Research Center for Model Organisms/SMOC, Shanghai 201203, China

**Keywords:** apigenin, combination, colorectal cancer, glycolysis, lonidamine

## Abstract

Colorectal cancer (CRC) is the second most prevalent cancer globally and remains a significant cause of cancer-related mortality. The limited efficacy and toxicities of conventional therapies underscore the urgent need for novel treatments. Lonidamine (LND), a synthetic indazole-3-carboxylic acid derivative, possesses anticancer properties, yet its clinical use is limited by toxic side effects. Apigenin (AP), a naturally occurring flavonoid present in a variety of fruits and vegetables, has been observed to enhance the efficacy of conventional chemotherapy regimens while mitigating associated side effects. In this study, we explored the potential synergistic anticancer effects and mechanisms of combining LND with AP in colon cancer cell lines MC38 and CT26. The results showed that LND and AP in combination synergistically inhibited the growth of colon cancer cells. In vitro, the combination therapy inhibited cell migration, induced cell cycle arrest in the G2/M phase, and promoted apoptosis by downregulating Bcl-2 and upregulating Bax expression. It disrupted glycolysis by reducing HK2 and GLUT1 expression, resulting in decreased glucose consumption and lactate production. Additionally, our findings suggested that the co-administration led to nucleotide depletion and disrupted NAD^+^ metabolism. The synergistic anticancer effect of LND combined with AP was also validated in MC38 tumor-bearing mice. These findings provide preliminary evidence that the combination of LND and AP may exert beneficial effects against CRC.

## 1. Introduction

Colorectal cancer (CRC) is one of the most common cancers worldwide and a leading cause of cancer death. In 2022, over 1.9 million new cases of CRC, including anal cancers, and 904,000 related deaths were estimated, accounting for approximately 10% of all cancer cases and deaths. Notably, CRC ranks third in incidence and second in mortality globally [1]. Currently, the primary therapeutic approaches for CRC include surgical removal, chemotherapy, radiation therapy, and immunotherapy [2]. However, these conventional therapies are often limited by severe side effects and the development of drug resistance [3]. These challenges highlight the urgent need for more effective and less toxic therapeutic strategies.

Rapidly proliferating tumor cells require not only accelerated energy production but also a substantial supply of metabolic intermediates to support their growth and biosynthetic demands. This drives metabolic reprogramming, characterized by a preference for glycolysis even in the presence of oxygen, a phenomenon called the Warburg effect [4,5]. By relying on glycolysis and altering mitochondrial function, cancer cells gain a metabolic advantage that promotes their rapid growth and survival. Given the important role of metabolic reprogramming in cancer cell proliferation, targeting these altered pathways has emerged as a promising therapeutic strategy. Lonidamine (LND), initially studied as a male contraceptive for its antispermatogenic effects [6], is now recognized for its ability to modulate tumor metabolism [7,8,9,10]. LND exerts its antitumor effects primarily by inhibiting key glycolytic enzymes such as hexokinase II, thereby disrupting to a disruption in the energy metabolism of cancer cells [11,12]. Furthermore, LND interferes with mitochondrial function, resulting in loss of mitochondrial membrane potential, increased oxidative stress and apoptosis [13].

Although LND has demonstrated anticancer potential, its clinical use is limited by low efficacy, toxicity, and resistance. Consequently, researchers have explored combination therapies to improve efficacy and minimize adverse effects. For example, combining LND with curcumin disrupts mitochondrial integrity, increases ROS, and promotes cancer cell death [14]. However, the clinical application of curcumin is limited by its poor bioavailability, caused by extensive metabolism and low intestinal absorption [15]. Similarly, LND combined with matrine inhibits HK-II expression, weakening cancer cell survival [16]. Nevertheless, the clinical applicability of matrine is significantly restricted by its severe side effects, including hepatotoxicity, neurotoxicity, and reproductive or developmental toxicity [17]. Co-administration of LND and cisplatin further lower cancer cells antiapoptotic activity by downregulating Bcl-2, boosting therapeutic effects [18], although cisplatin-associated toxicity remains a major concern. Collectively, these studies suggest that LND-based combinations can potentiate anticancer activity by targeting multiple pathways simultaneously; however, their clinical translation is often hampered by unfavorable pharmacological profiles and toxicity associated with the co-administered agents.

Previous studies have shown that the combination of lonidamine and quercetin, a flavonoid with diverse pharmacological effects, synergistically enhances cytotoxicity in cancer cells [19]. Apigenin (AP), another flavonoid compound, is widely present in various fruits and vegetables, such as celery, thyme, celeriac, chamomile, onions, oranges, and tea [20,21]. It exhibits a broad spectrum of biological activities, including antioxidant [22], anti-inflammatory [23], antibacterial [24], antiviral [25], and anticancer effects [26]. Numerous studies have demonstrated the antitumor effects of apigenin on various cancers, such as colorectal [27,28], lung [29], prostate [30], pancreatic [31], and breast cancers [32]. Importantly, AP has been shown to modulate multiple cancer-associated pathways, including cell cycle regulation, apoptosis induction, inhibition of tumor cell migration and invasion, and suppression of glycolysis, while possessing a favorable safety profile compared to many chemotherapeutic agents [33,34,35,36].

The antitumor potential of combining LND with AP in CRC has not yet been elucidated. Given the distinct yet complementary mechanisms of LND and AP, their combination may yield synergistic anticancer effects. Therefore, this study aims to investigate the combined antitumor effects of LND and AP in CRC and to explore the underlying molecular mechanisms, providing preliminary insights for subsequent research.

## 2. Materials and Methods

### 2.1. Cell Lines and Culture

Mouse colon cancer MC38 and CT26 cells were obtained from the Shanghai Model Organisms Center, Inc. (Shanghai, China). The HEK293T and LLC cell lines were maintained in our laboratory. The MC38, HEK293T and LLC cells were maintained in a high-glucose DMEM medium (HyClone, Logan, UT, USA), whereas the CT26 cells were cultured in RPMI-1640 (HyClone) supplemented with 10% fetal bovine serum and 1% penicillin-streptomycin, at 37 °C with 5% CO_2_.

### 2.2. Antibodies and Reagents

Western blotting and immunohistochemistry were performed using the following antibodies: Bax (Cat No. 60267-1-Ig, diluted 1:20,000 with antibody diluent), Bcl-2 (26593-1-AP, 1:3000), β-actin (20536-1-AP, 1:5000), and Ki67 (28074-1-AP, 1:4000) (Proteintech, Wuhan, China); HK2 (A0994, 1:2000), GLUT1 (A6982, 1:5000), and LDHA (A1146, 1:2000) (ABclonal, Wuhan, China). Lonidamine (LND; Meilune, Dalian, China) and apigenin (AP; Meilune) were dissolved using dimethyl sulfoxide (DMSO; Sigma-Aldrich, St. Louis, MO, USA). The Annexin V, FITC Apoptosis Detection Kit and the Cell Counting Kit-8 were purchased from Dojindo Laboratories (CCK8; Tokyo, Japan). The Cell Cycle Analysis Kit was purchased from the Beyotime Institute of Biotechnology (Shanghai, China). Lactic acid and glucose concentrations in the cell culture medium were evaluated using assay kits from Nanjing Jiancheng Bioengineering Institute (Nanjing, China), according to the provided instructions.

### 2.3. Cell Viability and Proliferation Assay

Cells (6 × 10^3^ cells/mL) were seeded into 96-well plates at a volume of 100 μL per well and left to incubate overnight. Subsequently, the cells were treated with 100 μL of medium containing LND, AP, or a combination of both for 48 h. Cell viability was assessed using a CCK-8 assay kit following the manufacturer’s instructions. Cell proliferation was determined by measuring the absorbance at 450 nm with a SpectraMax M5 plate reader (Molecular Devices, Sunnyvale, CA, USA). The calculation of cell viability was using the following equation: Cell Viability = [(As − Ab)/(Ac − Ab)] × 100%, where As means the absorbance of drug-treated cells, Ac is the absorbance of untreated control cells, and Ab is the absorbance of wells containing medium and CCK-8 reagent without cells and drugs.

### 2.4. Combination Index (CI) Analysis

Cells were seeded into 96-well plates and exposed to various concentrations of LND and AP, both individually and in combination. The synergistic therapeutic effect of the LND and AP combination was evaluated using the Chou–Talalay method [37]. The Chou-Talalay method for analyzing drug combinations utilizes the median-effect equation, which originates from the mass-action law principle. Using CompuSyn version 1.0 software (ComboSyn, Inc., New York, NY, USA), the combination index (CI) values were computed. The effects of the drug combination were evaluated using CI values: CI < 1 indicates synergy, CI = 1 indicates an additive effect, and CI > 1 indicates antagonism. An isobologram was produced with CompuSyn software to visually illustrate the synergistic, additive, and antagonistic interactions between the drugs.

### 2.5. Cell Migration Assay

A wound healing assay was employed to evaluate cell migration, following a previously described method [38]. The cells (4 × 10^5^ per well) were cultured in 6-well plates until they reached 80–90% confluence. A scratch was introduced into the cell monolayer using a 200 μL pipette tip, followed by two washes to remove debris. The cells were then cultured in serum-free medium containing LND and AP, both individually and in combination, for 12 and 24 h. Wound closure images were captured with an inverted microscope, maintaining consistent magnification and position. Cell migration rates at 12 and 24 h were evaluated using ImageJ version 1.54p software (U.S. National Institutes of Health, Bethesda, MD, USA).

### 2.6. Cell Cycle Analysis

Cells were seeded into 6-well plates at a concentration of 2 × 10^5^ cells/mL and exposed to the specified drugs for 24 h. After treatment, the cells were collected through trypsinization and washed two times with phosphate-buffered saline (PBS). They were then fixed in 70% ethanol at 4 °C for 2 h. After fixation, cells were stained with a solution containing propidium iodide (PI) and RNase A, and held at 37 °C in the absence of light for 30 min. Cell cycle distribution was analyzed using a CytoFLEX LX flow cytometer (Beckman Coulter, Brea, CA, USA).

### 2.7. Annexin V/PI Staining

The analysis of cell apoptosis was conducted using flow cytometry with an Annexin V, FITC Apoptosis Detection Kit. After treatment, cells were trypsinized and washed twice with cold PBS. Resuspend the cells at a density of 1 × 10^6^ cells/mL in 100 μL of 1 × Annexin V binding buffer. Add 5 μL AnnexinV-FITC and 5 μL Propidium Iodide (PI), then incubate in the dark at room temperature for 15 min. Add 400 μL of 1X Annexin V binding buffer after incubation to make the final volume 500 μL. Filter the cells through a 40 μm mesh and analyze using a CytoFLEX LX flow cytometer within an hour. The percentage of apoptotic cells was determined as the sum of Annexin V^+^/PI^−^ (early apoptosis) and Annexin V^+^/PI^+^ (late apoptosis).

### 2.8. Real-Time Quantitative Reverse Transcription PCR (RT-qPCR)

Total RNA was extracted from collected cells using Trizol (TIANGENBiotech, Beijing, China) according to the manufacturer’s instructions. The PrimeScript RT reagent Kit with gDNA Eraser (Takara, Shiga, Japan) was used to synthesize cDNA from 1 μg of RNA. Quantitative real-time PCR was conducted using the TB Green Premix Ex Taq II (Takara). The relative gene expression levels were assessed and quantified through the ΔΔCt method. The data were standardized to the β-actin level, and Table S1 shows the primer sequences.

### 2.9. Western Blot Analysis

Protein samples were harvested using RIPA buffer (Epizyme, Shanghai, China) with the addition of a protease inhibitor cocktail (Beyotime) and Phenylmethanesulfonyl fluoride (PMSF; Beyotime). The samples were placed in protein loading buffer (Epizyme) and boiled at 95 °C for 5 min. Proteins were separated using sodium dodecyl sulfate-polyacrylamide gel electrophoresis (SDS-PAGE) and subsequently transferred to polyvinylidene difluoride (PVDF) membranes (Millipore, Billerica, MA, USA). The membranes were incubated with primary antibodies overnight at 4 °C. After washing with 1×Tris-buffered saline containing 0.1% Tween20 (1×TBST), they were incubated with the appropriate horseradish peroxidase (HRP)-conjugated secondary antibody at room temperature. Finally, membranes were visualized using an ECL Western blotting Detection Reagent (Beyotime). All protein levels were measured by Western blot, normalized to β-actin, and then normalized to the control group.

### 2.10. Metabolite Measurement Using Ultra-Performance Liquid Chromatography–Tandem Mass Spectrometry (UPLC-MS/MS)

Cells in the logarithmic growth phase (5 × 10^6^ cells) were collected and washed twice with ice-cold phosphate-buffered saline (PBS). The cell pellet was then resuspended in 1 mL of 80% methanol pre-chilled to −80 °C, followed by two freeze–thaw cycles using liquid nitrogen. The samples were shaken at 1500 rpm for 30 min at 4 °C. Following a 15 min centrifugation at 14,000 rpm and 4 °C, the supernatant was separated and stored at −80 °C for later analysis. Metabolite analysis was performed on a UPLC-MS/MS platform equipped with an Agilent 1290 liquid chromatograph (Agilent Technologies, Palo Alto, CA, USA) and an AB Sciex QTRAP 6500plus mass spectrometer (AB Sciex, Framingham, MA, USA). Chromatographic separation was achieved using a Merck ZIC^®^-pHILIC column (5 μm, 150 × 2.1 mm, Merck, Darmstadt, Germany). Data acquisition was performed using Analyst 1.7.1 software (AB Sciex) in multiple reaction monitoring (MRM) mode. Data were analyzed using Sciex OS 2.1 software (AB Sciex), including retention time (RT) alignment and background noise reduction based on blank runs.

### 2.11. In Vivo Experiment

All animal studies involved in this study were conducted following the ethical guidelines of the Institutional Animal Care and Use Committee of Tongji University (TJAB06224101). The approval date was 9 December 2024. Female C57BL/6 mice (5–6 weeks old) were obtained from the Shanghai Model Organisms Center (Shanghai, China) and housed in a pathogen-free environment at the experimental animal center. A colorectal cancer mouse model was constructed by subcutaneously injecting MC38 cells (1 × 10^6^ cells/mouse). Mice were randomized into four groups (5 mice per group) with the mean tumor volume for each group being 50–100 mm^3^ as follows: (i) Vehicle control (methylcellulose/Tween 80, intraperitoneal injection [ip], every 2 d); (ii) Lonidamine (30 mg/kg, ip, every 2 d); (iii) Apigenin (15 mg/kg, ip, every 2 d); and (iv) Combination of Lonidamine (30 mg/kg) and Apigenin (15 mg/kg) (ip, every 2 d). Tumor size was measured using calipers, and tumor volumes were calculated using the formula: Volume = length × width^2^ × 0.5. After the treatment, the mice were euthanized, and tumor tissues along with major organs were collected and fixed in 4% paraformaldehyde. Subsequently, these tissues are prepared for immunohistochemical staining.

### 2.12. Immunohistochemical (IHC) Staining

The isolated tumor tissues were treated with 4% paraformaldehyde fixation and then embedded within paraffin. The tissues were dehydrated, paraffin embedded and then cut into 5-μm-thick sections. Immunohistochemical staining was conducted according to standard methods, including the following steps: dewaxing of paraffin sections, antigen retrieval, blocking endogenous peroxidase, serum closure, incubation with primary antibody (anti-Ki67), incubation with secondary antibody, DAB color development, restaining nuclei, dewatering and sealing. The sections were observed using an optical microscope (Olympus, Tokyo, Japan).

### 2.13. Statistical Analysis

Statistical significance between groups was assessed using *t*-test or ANOVA, with a *p*-value < 0.05 considered statistically significant. For comparisons between two groups, data normality was assessed using the Shapiro–Wilk test. Normally distributed data were analyzed using an unpaired *t*-test; if the assumption of equal variances was violated, Welch’s correction was applied. Non-normally distributed data were evaluated using the Mann–Whitney test. For comparisons among three or more groups, ANOVA followed by Dunnett’s post hoc test was employed. **** *p* ≤ 0.0001, *** *p* ≤ 0.001, ** *p* ≤ 0.01, * *p* ≤ 0.05, ns, not significant. Results are shown as the mean ± standard deviation (SD) from at least three independent experiments. All results were analyzed using GraphPad Prism 9.4.1 software (San Diego, CA, USA).

## 3. Results

### 3.1. Lonidamine and Apigenin Co-Treatment Synergistically Inhibits Proliferation and Migration of Colon Cancer Cells

Previous studies have shown that LND is effective at concentrations of 20–300 μM, while AP is typically used at 5–50 μM in various cancer cell lines [28,39,40,41,42,43,44,45,46,47]. Based on these reports, we selected appropriate dose ranges for subsequent assays. To evaluate the effect of the combination of LND and AP on cell proliferation, MC38 cells were treated with varying concentrations of LND (40–180 µM) and AP (4–18 µM) separately for 48 h. In contrast, CT26 cells were treated with LND (40–180 µM) and a higher range of AP concentrations (10–45 µM) separately, reflecting their differing sensitivities to the drugs. The results indicated that treatment with either LND or AP individually led to a dose-dependent reduction in cell viability in both MC38 and CT26 cell lines (Figure S1A–D).

**Figure 1:**
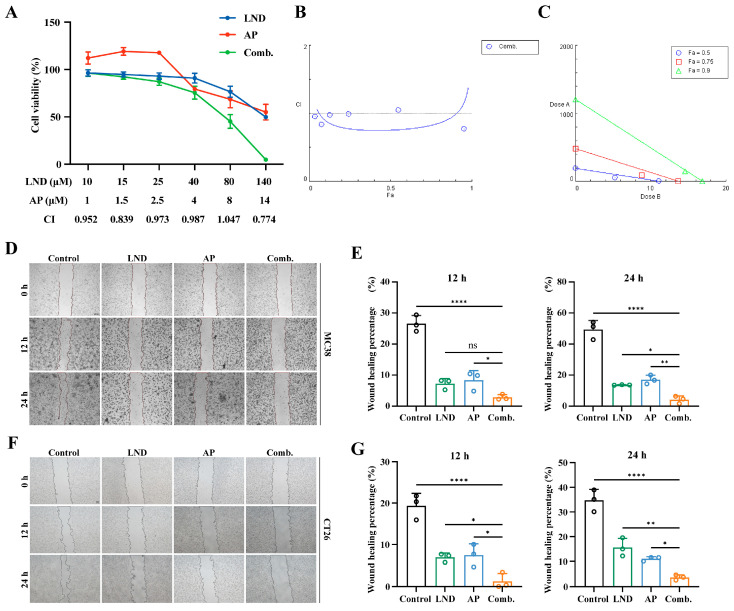
The combination of lonidamine (LND) and apigenin (AP) synergistically inhibits the proliferation and migration of colon cancer cells. (**A**) MC38 cells were exposed to varying concentrations of LND and/or AP for 48 h, and cell viability was measured relative to the untreated control. (**B**) Combination index (CI) plot of (**A**). (**C**) Isobologram of (**A**). (**D**) Cell migration of MC38 cells cotreated with LND (140μM) and AP (14μM) at 0 h, 12 h, and 24 h. (**E**) Quantitative analysis of (**D**). (**F**) Cell migration of CT26 cells cotreated with LND (160μM) and AP (40μM) at 0 h, 12 h, and 24 h. (**G**) Quantitative analysis of (**F**). All data are from at least three independent experiments. Results are presented as mean ± SD. Data were analyzed by one-way ANOVA. **** *p* ≤ 0.0001, ** *p* ≤ 0.01, * *p* ≤ 0.05, ns, not significant.

Furthermore, the efficacy of the combined treatment with LND and AP was assessed using various drug concentration ratios in the MC38 cell line. As shown in Figure S1E, the combination treatment of 140 µM LND and 14 µM AP at a 10:1 ratio significantly inhibited cell proliferation more effectively than either treatment alone (Figure S1F). The isobologram analysis also demonstrated a synergistic interaction between 140 µM LND and 14 µM AP when combined at a 10:1 ratio (Figure S1G). We further assessed the impact of the 10:1 LND and AP combination on cell viability in MC38. The results indicated that the combination of the two drugs exhibited a significant synergistic or additive effect, with the most pronounced synergistic effect observed at an LND:AP ratio of 140:14 (Figure 1A,B). The isobologram analysis revealed the synergistic interaction between LND and AP at fraction affected (Fa) levels of 0.5, 0.75, and 0.9 (Figure 1C). Thus, subsequent experiments employed 140 µM LND and 14 µM AP. In the CT26 cell line, we similarly observed a pronounced synergistic effect from the combination of the two drugs (Figure S1H,I). In contrast, the HEK293T cell line showed less pronounced inhibition of cell proliferation with both single-drug and combination treatments compared to MC38 and CT26 (Figure S1J).

To assess the impact of LND and AP on cell migration, a wound-healing assay was performed in both MC38 and CT26 cells. As shown in Figure 1D–G, the combination treatment of LND and AP was more effective than monotherapy in inhibiting cell migration at both 12 and 24 h. This led to a larger scratched area, confirming the synergistic effect of LND and AP.

### 3.2. Combination Treatment of Lonidamine and Apigenin Induced Apoptosis and Cell Cycle Arrest

**Figure 2:**
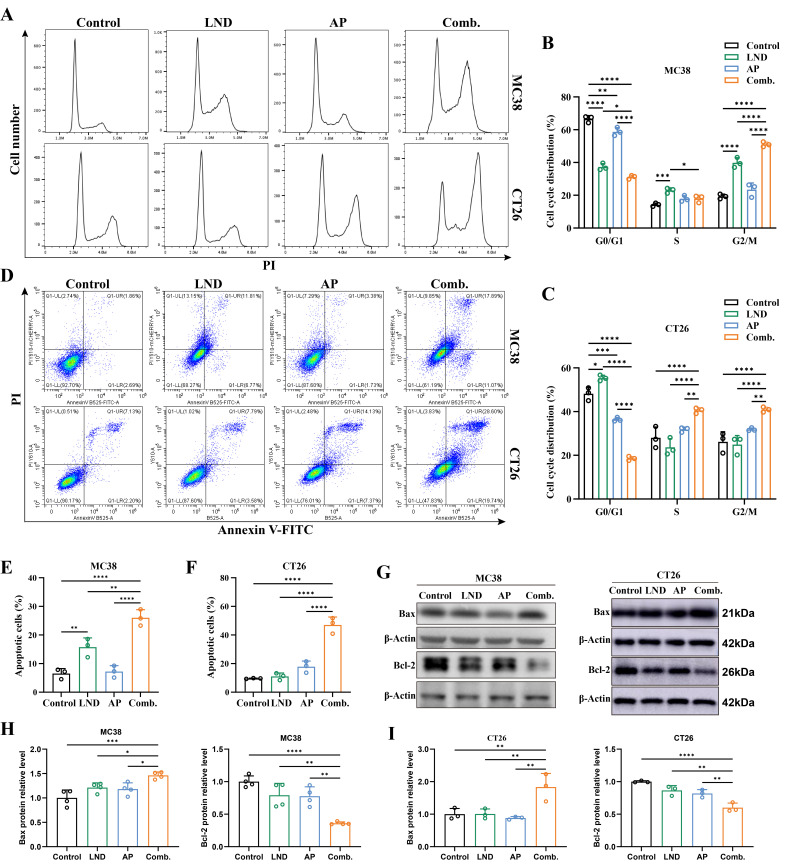
The combination of LND and AP induces G2/M cell cycle arrest and significantly increases apoptosis rates. (**A**) Cell cycle distribution of MC38 cells (140 μM LND, 14 μM AP, or both) and CT26 cells (160 μM LND, 40 μM AP, or both) was analyzed by flow cytometry. (B, C) The percentages of cells in the G0/G1, S, and G2/M phases were quantified for MC38 (**B**) and CT26 (**C**). Data were analyzed by two-way ANOVA. (**D**) Apoptosis in MC38 and CT26 cells determined by flow cytometry following FITC Annexin V/PI staining. (**E**,**F**) Quantitative of apoptotic MC38 (**E**) and CT26 (**F**) cells. (**G**) Western blot analysis of Bax and Bcl-2 protein expression in MC38 and CT26 cells, with β-actin as a loading control. (**H**,**I**) Quantitative analysis of (**G**). All data are from at least three independent experiments. Results are presented as mean ± SD, with statistical significance determined by one-way ANOVA as follows: **** *p* ≤ 0.0001, *** *p* ≤ 0.001, ** *p* ≤ 0.01, * *p* ≤ 0.05.

To further elucidate the mechanism behind the synergistic antiproliferative effect of LND and AP, we subsequently employed flow cytometry to evaluate the impact of their combination on cell cycle progression and apoptosis induction. In both MC38 and CT26 cells, the combination treatment of LND and AP significantly increased the proportion of cells in the G2/M phase while simultaneously reducing the G0/G1 phase population compared to controls and cells treated with either agent alone (Figure 2A–C). In MC38 cells, both LND and AP treatments reduced the G0/G1 phase fraction, while LND alone increased the S and G2/M phase fractions (Figure 2A,B). Conversely, in CT26 cells, LND treatment increased the G0/G1 phase fraction, while AP treatment decreased it (Figure 2A,C). These results indicated that the combination of LND and AP may exert a more pronounced effect on cell cycle progression than either agent alone.

The Annexin V-FITC assay showed that LND treatment alone induced moderate apoptosis (16%) in MC38 cells, while AP treatment alone did not significantly induce apoptosis, showing no statistical difference compared to the control group (Figure 2B). In CT26 cells, treatment with either LND or AP alone did not result in a significant increase in apoptosis compared to the control group. Notably, co-treatment with LND and AP significantly increased apoptosis, reaching 26% in MC38 cells (*p* < 0.0001, Figure 2D,E) and 47% in CT26 cells (*p* < 0.0001, Figure 2D,F). The results were further validated by Western blotting, which showed a significant increase in Bax expression and a marked decrease in Bcl-2 expression, both critical protein markers associated with apoptosis (Figure 2G–I). Collectively, these results indicated that the combination of LND and AP markedly promotes apoptosis in mouse colorectal cancer cells, which is one of the mechanisms behind their synergistic antiproliferative effect.

### 3.3. Inhibition of Glycolytic Pathways and Energy Production

**Figure 3:**
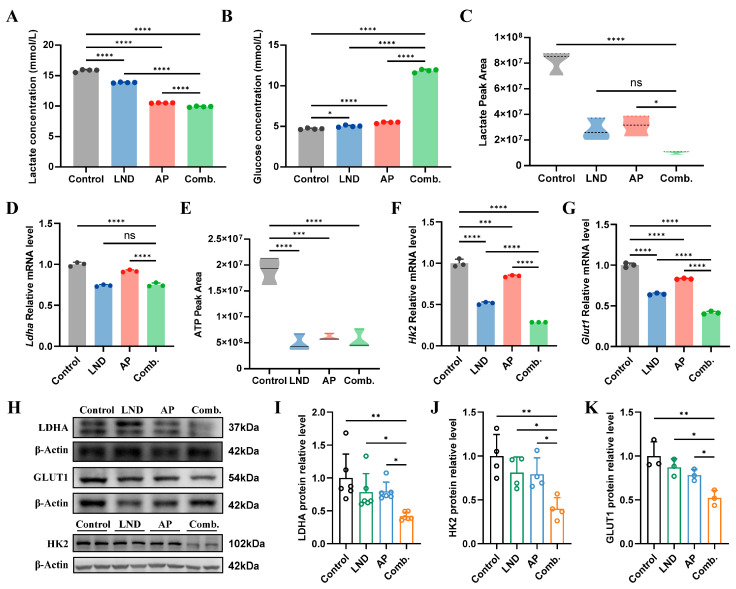
Inhibition of Glycolytic Pathways and Energy Production by the combination of LND and AP. (**A**,B) Lactate (**A**) and glucose (**B**) levels in the culture medium from MC38 cells treated with 140 μM LND, 14 μM AP, or both, were quantified using assay kits. (**C**) Intracellular lactate content was determined by UPLC-MS/MS. (**D**) *LDHA* mRNA expression was assessed by RT-qPCR. (**E**) Intracellular ATP content was measured by UPLC-MS/MS. (**F**) *Hk2* and (**G**) *Glut1* mRNA expression levels were determined by RT-qPCR. (**H**–**K**) Western blot and quantitative analysis of LDHA, HK2 and GLUT1 protein levels in MC38 cells. All data are from at least three independent experiments and are presented as mean ± SD. Data were analyzed using one-way ANOVA. **** *p* ≤ 0.0001, *** *p* ≤ 0.001, ** *p* ≤ 0.01, * *p* ≤ 0.05, ns, not significant.

Building on previous studies emphasizing the critical role of glucose metabolism in the anticancer mechanisms of LND and AP [35,48], we sought to examine the bioenergetic alterations induced by their combination, aiming to uncover the molecular mechanisms driving their synergistic effects. First, we investigated whether the combination of LND and AP impacts lactate production and glucose consumption, two key indicators of the Warburg effect in cancer cells, using lactate and glucose assay kits. Both LND and AP individually reduced lactate levels and increased glucose content in the medium of MC38 cells (Figure 3A,B). Notably, their combination exerted the most pronounced effects on both lactate and glucose levels (Figure 3A,B). The UPLC-MS/MS results revealed a significant decrease in intracellular lactate levels following the combination treatment compared to the control group (Figure 3C). However, no significant difference in intracellular lactate levels was observed between the combination group and the LND group (Figure 3C). This finding was further corroborated by RT-qPCR (Figure 3D) and Western blot (Figure 3H,I) analysis, which showed consistent trends in the expression of lactate-related gene *Ldha*. Furthermore, LND, AP, and their combination significantly reduced intracellular ATP levels in MC38 cells (Figure 3E).

GLUT1 and HK2 are essential in regulating the initial stages of glycolysis, including glucose uptake and the first rate-determining step [49]. LND has been reported to inhibit the rate-limiting glycolytic enzyme HK2, thereby disrupting the energy supply required for tumor cell proliferation [50]. Previous studies have demonstrated that AP can suppress the expression of *Hk2* and *Glut1* mRNA in various cancer cell lines [35,51]. In our study, we observed that both LND and AP independently reduced *Hk2* and *Glut1* expression. Moreover, their combined administration resulted in a more significant inhibitory effect on these key metabolic regulators (Figure 3F–H,J,K). The results showed that glycolytic inhibition was observed following the combined treatment with LDN and AP, along with lowered intracellular energy levels.

### 3.4. Impact of Lonidamine and Apigenin on Nucleotide Depletion and NAD Disruption

**Figure 4:**
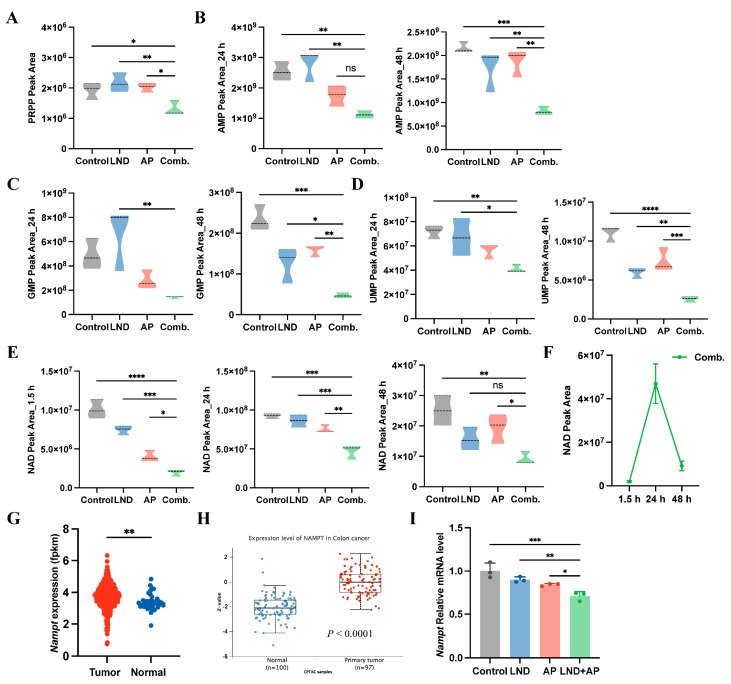
Impact of LND and AP on nucleotide depletion and NAD disruption. (**A**–**E**) PRPP (**A**), AMP (**B**), GMP (**C**), UMP (**D**), and NAD (**E**) levels in MC38 cells (140 μM LND, 14 μM AP, or both) were measured by UPLC-MS/MS. (**F**) Time-dependent changes in NAD levels were analyzed in the LND and AP combination treatment group. (**G**,**H**) *Nampt* mRNA expression in normal colon tissue and colon cancer tissue was analyzed using TCGA data (**G**), and protein expression was analyzed using CPTAC data (**H**). Data were analyzed by *t*-test. ** *p* ≤ 0.01. (**I**) *Nampt* mRNA levels in MC38 cells were measured by RT-qPCR. All data are from at least three independent experiments. Data were analyzed by one-way ANOVA. **** *p* ≤ 0.0001, *** *p* ≤ 0.001, ** *p* ≤ 0.01, * *p* ≤ 0.05, ns, not significant.

Cancer cells reprogrammed glucose metabolism to accelerate de novo nucleotide biosynthesis, a hallmark of their metabolic adaptation [52]. ATP deficiency directly limits the synthesis of 5-phosphoribosyl-1-pyrophosphate (PRPP), a critical precursor for purine and pyrimidine biosynthesis. Our analysis revealed that intracellular PRPP levels in MC38 cells were unaffected by individual administration of LND or AP (Figure 4A). However, the combination of LND and AP caused a marked reduction in PRPP levels compared to both the untreated control group and the single-agent treatments (Figure 4A). Notably, the combination of LND and AP significantly reduced the levels of purine intermediates (AMP and GMP) and the pyrimidine intermediate (UMP) at 24 and 48 h (Figure 4B–D).

PRPP plays a critical role not only as a key intermediate in de novo nucleotide biosynthesis but also as a co-substrate in the terminal steps of de novo NAD synthesis [53]. Notably, we observed that the combined treatment of LND and AP resulted in a significant reduction in NAD levels (Figure 4E), with NAD levels exhibiting a transient increase followed by a subsequent decline over time (Figure 4F). Nicotinamide phosphoribosyl transferase (NAMPT), is a key enzyme in NAD metabolism, catalyzing the rate-limiting conversion of nicotinamide (NAM) to nicotinamide mononucleotide (NMN), which supports NAD production [54]. Differential expression analysis using The Cancer Genome Atlas (TCGA) and Clinical Proteomic Tumor Analysis Consortium (CPTAC) datasets revealed significantly higher NAMPT mRNA and protein levels in colon cancer tissues compared to normal tissues (Figure 4G,H). Additionally, we observed that LND and AP combination treatment reduced *Nampt* mRNA levels in MC38 cells (Figure 4I). These results suggest that the combination therapy of LND and AP potentially interferes with both de novo nucleotide and NAD biosynthesis pathways.

### 3.5. Lonidamine Combined with Apigenin Suppressed Tumor Growth In Vivo

**Figure 5:**
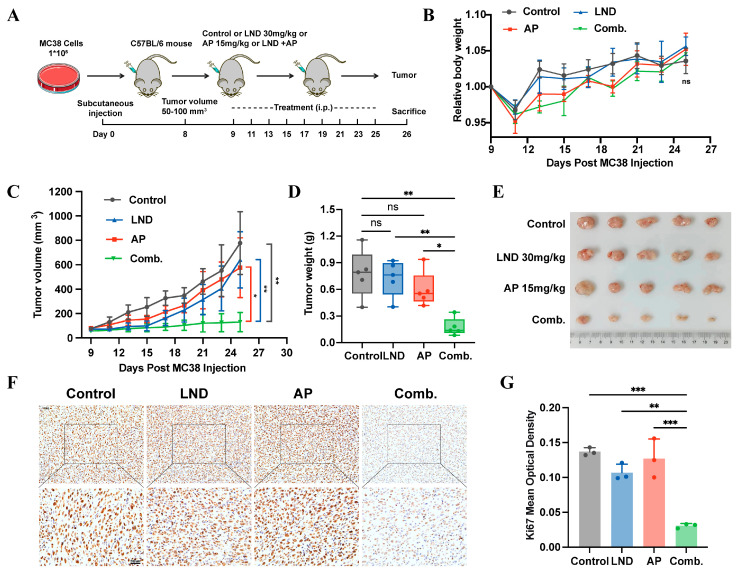
LND combined with AP suppressed tumor growth in vivo. (**A**) Schematic illustration of the treatment regimen. (**B**) Body weight of mice treated with 30 mg/kg LND, 15 mg/kg AP, or a combination of both. (**C**) Tumor volume variation curves for mice in different treatment groups. (**D**) Tumor weight (*n* = 5 for each group). (**E**) Digital photographs of the tumors dissected from mice in different groups. (**F**,**G**) Immunohistochemistry for detecting Ki67 in tumor tissue. Data were analyzed by one-way ANOVA. *** *p* ≤ 0.001, ** *p* ≤ 0.01, * *p* ≤ 0.05, ns, not significant.

To further identify the anticancer effects of combining LND and AP in vivo, we established a mouse CRC model using MC38 cells (Figure 5A). One of the primary concerns with an increased number of drugs is the potential for side effects. Lonidamine (50 mg/kg, i.p.) has been demonstrated to enhance the therapeutic efficacy of cisplatin in a murine lung cancer model. Notably, its effects are significantly amplified when combined with systemic hyperthermia, leading to pronounced tumor growth inhibition [55]. Apigenin treatment (50 mg/kg, i.p.) significantly suppressed the growth of solid Ehrlich carcinoma tumors and exhibited synergistic radiosensitizing effects when combined with γ-irradiation [56]. Based on these findings, we established a series of concentration gradients for LND and AP (Figure S1A–F). The results revealed that LND (30 mg/kg) and AP (15 mg/kg) alone did not exhibit significant tumor inhibitory effects. However, when they are combined, a strong tumor-suppressing effect is demonstrated. Moreover, in terms of body weight, the combined use of the two drugs does not cause obvious side effects (Figure 5B–E). To further investigate the impact of the LND and AP combination on tumor growth, Ki67 expression levels were assessed via immunohistochemistry. The results revealed a marked decrease in Ki67 expression (Figure 5F,G), indicating that the combination treatment effectively suppressed cell proliferation in vivo. Collectively, these findings demonstrate that the combination of LND and AP exhibits significant synergistic antitumor effects in MC38 mouse models.

## 4. Discussion

The role of glycolysis in cancer progression has attracted considerable attention, with the Warburg effect, defined by the preferential utilization of glycolysis for energy production in cancer cells, emerging as a hallmark of cancer metabolism [9]. However, therapies targeting glycolytic pathways have not achieved anticipated outcomes in cancer patients, likely due to cancer cell resistance or dose-limiting toxicities [57]. Lonidamine, a derivative of indazole-3-carboxylic acid, disrupts glycolysis by inhibiting key enzymes, such as hexokinase and lactate transporters. By interfering with these metabolic processes, lonidamine impairs cancer cell growth and survival through disruption of energy production and alteration of intermediate metabolite balance. However, its clinical application is limited by adverse effects and limited anticancer efficacy when used as a monotherapy [10]. Apigenin, a natural flavonoid, has emerged as a promising chemosensitizer and chemopreventive agent [58]. When combined with other drugs, apigenin can enhance their therapeutic efficacy while reducing the risk of side effects. In this study, we investigated the antitumor effects of the combination of LND and AP in both murine colorectal cancer models and colorectal cancer cell lines. Through concentration gradient experiments conducted both in vitro and in vivo, we identified the optimal concentrations of LND and AP (Figures S1A–D and S2). Compared to treatments with single agents, the combination therapy exhibited a more pronounced inhibitory effect on cell proliferation (Figure 1), promoted apoptosis and cell cycle arrest (Figure 2), suppressed glycolysis (Figure 3), and induced nucleotide depletion as well as NAD disruption (Figure 4). Interestingly, a slight increase in cell viability was observed at lower concentrations of AP (Figure 1A). This phenomenon may be explained by a hormetic response, wherein low doses of flavonoids primarily act as antioxidants, thereby alleviating oxidative stress and promoting cell survival and proliferation [59].

Glucose transporter 1 (GLUT1) is essential for glucose uptake and energy metabolism, particularly in the aerobic glycolysis pathway of tumor cells, a process closely linked to tumor progression and malignancy. Yanhong Sun et al. conducted systematic library screening and biological evaluations, identifying GLUT1 as a key target of apigenin and demonstrating that apigenin effectively suppresses GLUT1 expression [60]. Additionally, other studies have reported that apigenin reduced GLUT1 expression and inhibited the growth of ACC-2 tumor cells [61]. Our results highlighted that LND and AP alone suppress GLUT1 expression in MC38 cells, with a more pronounced effect observed when used in combination (Figure 3G,H,K). HK2 serves as a key regulator in aerobic glycolysis, driving the initial glucose phosphorylation step. In this study, the combined treatment with LND and AP significantly downregulated HK2 expression, which was accompanied by changes consistent with reduced aerobic glycolysis (Figure 3F,H,J). Altered glucose metabolism is marked by increased glucose uptake and elevated lactate production [62,63]. Our results further indicate that the combination of LND and AP reduces glucose uptake and decreases lactate production (Figure 3A–D).

Tumor cells depend on the efficient synthesis of nucleotides to sustain their rapid proliferation [64]. Phosphoribosyl pyrophosphate (PRPP) serves as a pivotal metabolite in various metabolic pathways, playing a key role in the production of purine and pyrimidine nucleotides, as well as NAD synthesis [65,66]. In this study, we observed that co-treatment with LND and AP significantly downregulated PRPP levels in mouse colorectal cancer cells, accompanied by reductions in nucleotide and NAD biosynthesis (Figure 4).

Our experiments with CT26 cells utilized LND at 160 μM and AP at 40 μM, concentrations approximating their IC50 values from proliferation assays (Figure S1C,D), which can differ among cell lines. For MC38 cells, the optimal synergistic effect was observed with a 10:1 ratio of LND (140 μM) to AP (14 μM) (Figure 1A–F), while in CT26 cells, a 4:1 ratio (LND 160 μM: AP 40 μM) showed substantial synergy in suppressing tumor cell proliferation (Figure S1E,F). Consistent with the in vitro findings, in vivo experiments revealed that co-treatment with LND and AP markedly suppressed tumor growth in MC38 tumor-bearing mice (Figure 5). Furthermore, the combination therapy also exhibited antitumor efficacy in C57BL/6 mice inoculated with LLC (Lewis lung carcinoma) cells (Figure S3), which could indicate a potential broader applicability.

Additionally, we found that the combined treatment of LND and AP significantly reduced acetyl-CoA levels in MC38 cells (Figure S4A–C), indicating potential inhibition of the tricarboxylic acid (TCA) cycle and subsequent energy depletion. This metabolic dysregulation may further impair lipid biosynthesis, thereby restricting cell membrane formation and signal transduction, ultimately contributing to the suppression of tumor growth.

Although our findings provide preliminary evidence regarding the antitumor effects of LND and AP co-treatment, the study has certain limitations, and further systematic research is needed to fully elucidate its mechanisms and functional significance. The in vitro work was confined to murine CRC cell lines (MC38 and CT26), which, despite their widespread use, may not fully recapitulate the molecular and metabolic characteristics of human CRC. Additionally, standard culture conditions (e.g., high glucose) may not reflect the tumor microenvironment, potentially affecting drug efficacy. In vivo experiments utilized MC38 tumor-bearing mice, whose metabolic and immune systems differ significantly from humans, limiting translational potential. Validation in human-derived models, such as patient-derived xenografts (PDX), is necessary to better mimic the human tumor microenvironment. Future studies should employ human CRC cell lines and preclinical models such as patient-derived xenografts (PDX) to facilitate the translation of murine findings to clinical applications. In addition, incorporating rescue experiments via NAD supplementation or direct modulation of NAMPT will help confirm the role of NAD metabolism and clarify the mechanisms driving the synergistic effects of LND and AP co-treatment.

Collectively, our results demonstrate that the combination of LND and AP synergistically suppresses the proliferation and migration of murine colorectal cancer cells in vitro and effectively inhibits tumor growth in vivo in tumor-bearing mice. Further mechanistic investigations are needed to clarify the molecular basis of the observed synergy, which may provide deeper insight into the underlying biological processes.

## Data Availability

The original contributions presented in this study are included in the article/supplementary material. Further inquiries can be directed to the corresponding author(s).

## Supplementary Materials

The following supporting information can be downloaded at: https://www.mdpi.com/article/10.3390/cimb47100825/s1.

## Abbreviations

AP	Apigenin
Comb.	Combination
CRC	Colorectal cancer
GLUT1	Glucose transporter 1
HK2	Hexokinase 2
LDHA	Lactate dehydrogenase A
LND	Lonidamine
PDX	Patient-derived xenografts
PRPP	Phosphoribosyl pyrophosphate
