# Supplementary material for: The Interventional Effects and Mechanisms of Lonidamine in Combination with Apigenin on Colorectal Cancer
Source: Curr Issues Mol Biol. 2025 Oct 8;47(10):825. doi: 10.3390/cimb47100825 (PMC12563077; doi:10.3390/cimb47100825)
Supplement: Supplementary file 1 [file cimb-47-00825-s001.zip › cimb-3860406-supplementary.pdf]

Supplementary Materials

1 Supplementary Figures and Tables

1.1 Supplementary Tables

Table S1. Primer sequences table.

Gene	Forward primer	Reverse primer	Main Accession No.
Hk2 (mouse)	F 5′- TCAAAGTGACGGTGGGCGTG-3′	R 5′- CACGTCACATTTCGGAGCCAG-3′	NM_013820
Glut1 (mouse)	F 5′- GGATGTCCTATCTGAGCATCGTG- 3′	R 5′- GCCAGCCACAGCAATAGCA-3′	NM_011400
Ldha (mouse)	F 5′- GTAACTGCGAACTCCAAGCTG-3′	R 5′- GCTTGCAGTGTGGACTGTAC-3′	NM_001136069
Nampt (mouse)	F 5′-CATTCAAGGAGATGGCGTGG-3′	R 5′-CATAGCTGCACTTGAAGGAGCA-3′	NM_021524
β-actin (mosue)	F 5′-GCTGACAGGATGCAGAAGGA-3′	R 5′- GCTGGAAGGTGGACAGTGAG-3′	NM_007393

Note: All primers were synthesized by GENEWIZ Bio Inc. (Suzhou, China).

1.2 Supplementary Figures

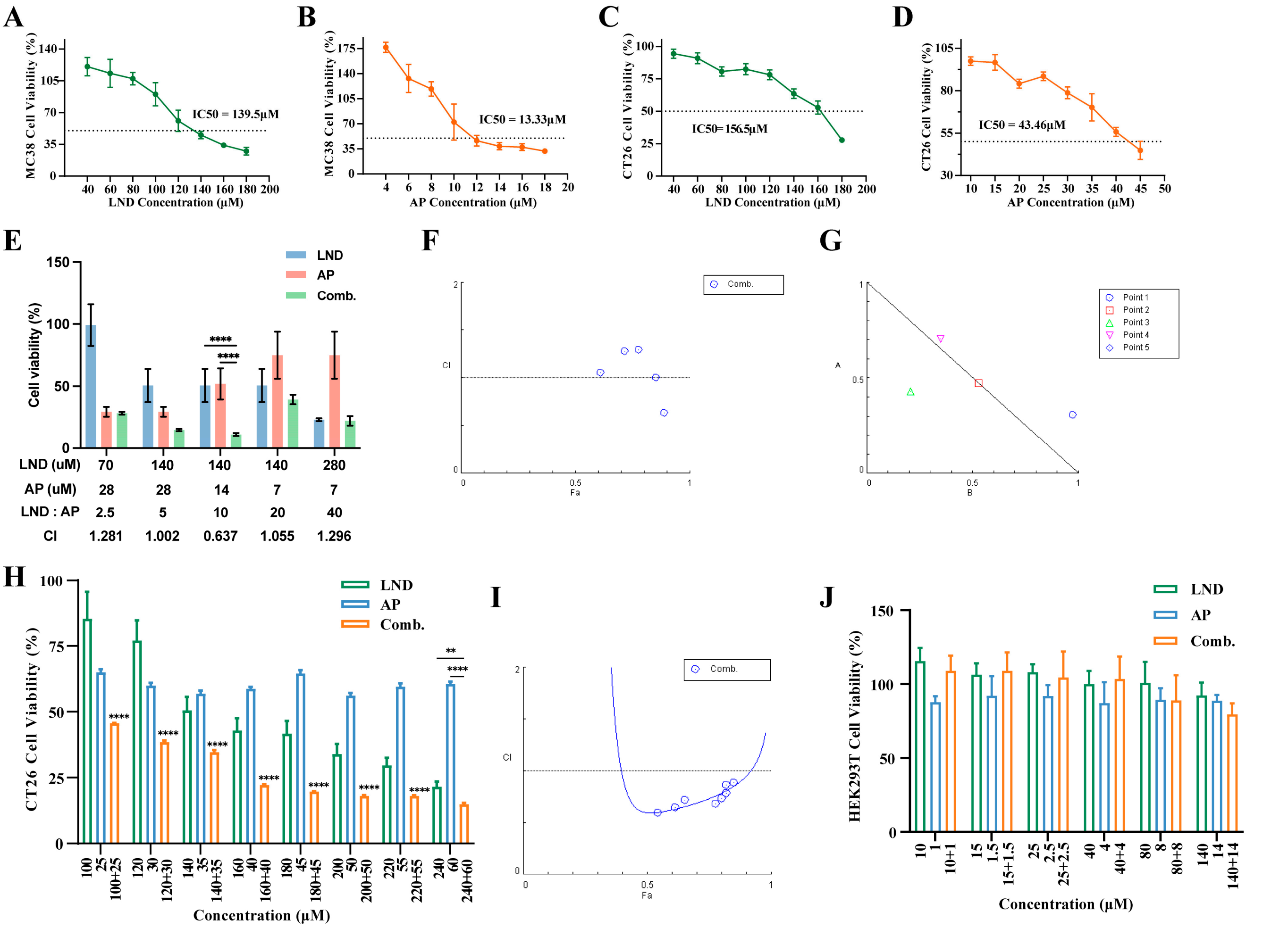

Figure S1. Effects of lonidamine (LND) and apigenin (AP) on cell viability. (**A**,**B**) MC38 cells were treated with different concentrations of lonidamine (**A**) and apigenin (**B**), and cell viability was measured relative to the untreated control. (C,D) CT26 cells were treated with different concentrations of lonidamine (**C**) and apigenin (**D**), and cell viability was measured. (**E**) MC38 cells were cultured with different concentrations and ratios of LND and AP, either alone or in combination,

for 48 hours, and cell proliferation was determined. (**F**) Combination index (CI) plot. The CI was calculated using CompuSyn software, where CI values < 1, = 1, and > 1 indicate synergism, an additive effect, and antagonism, respectively. (**G**) Isobologram of the combination of two drugs. Data points on the diagonal line indicate additive effects; points in the lower left indicate synergism, and points in the upper right indicate antagonism. Point 5 corresponds to a CI value of 1.296, which exceeds the upper right range and cannot be displayed. (**H**) CT26 cell proliferation with an LND to AP ratio of 4:1. (**I**) Combination index (CI) plot of (**H**). (**J**) HEK293T cells were treated with different concentrations of lonidamine and apigenin, and cell viability was measured. Results are presented as mean ± SD. **** $p \leq 0.0001$, ** $p \leq 0.01$ vs. combination group (Comb.).

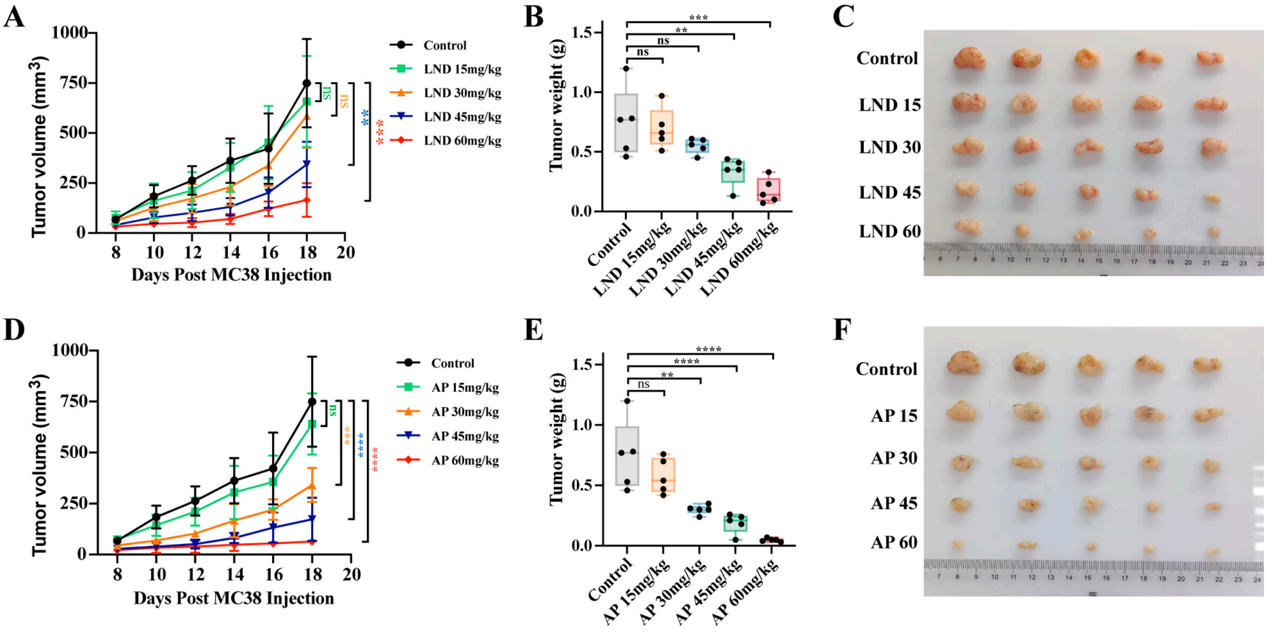

Figure S2. Lonidamine and apigenin inhibit tumor growth in MC38 tumor-bearing C57BL/6 mice. (**A**,**D**) Tumor volume variation curves were generated to evaluate the effects of different concentrations of lonidamine (**A**) and apigenin (**D**) on tumor growth. (**B**,**E**) Tumor weight was measured after treatment with different concentrations of lonidamine (**B**) and apigenin (**E**). (**C**,**F**) Digital photographs of tumors treated with different concentrations of lonidamine (**C**) and apigenin (**F**). Results are presented as mean ± SD. **** $p \leq 0.0001$, *** $p \leq 0.001$, ** $p \leq 0.01$, ns, not significant.

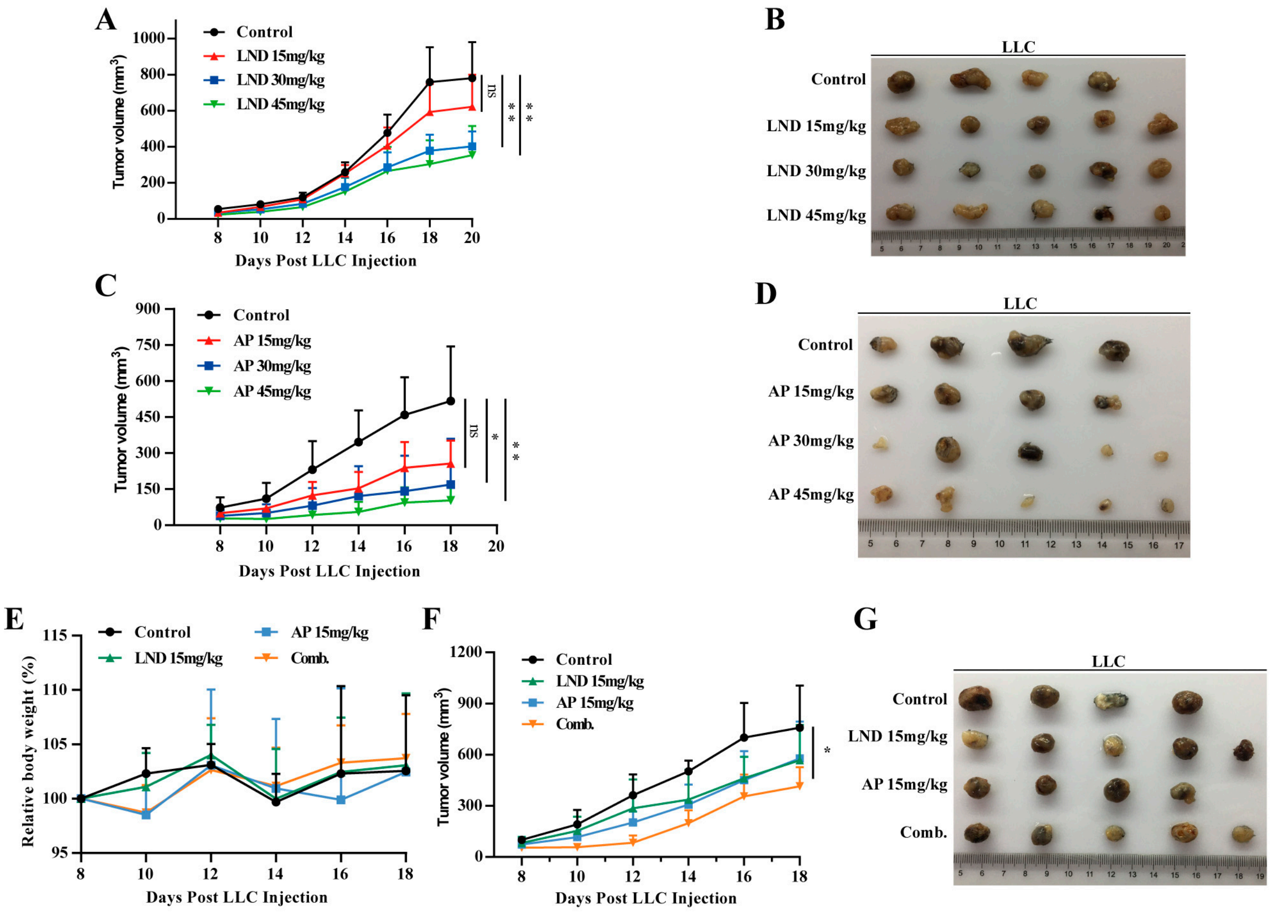

Figure S3. Lonidamine and apigenin inhibit tumor growth in LLC tumor-bearing C57BL/6 mice. (A,C) Tumor volume variation curves were generated to evaluate the effects of different concentrations of lonidamine (**A**) and apigenin (**C**) on tumor growth. (**B**,**D**) Digital photographs of tumors treated with different concentrations of lonidamine (**B**) and apigenin (**D**). (**E**) Body weight of mice. (**F**) Tumor volume variation curves for mice in different treatment groups (note that the control group in (**C**,**F**) was derived from the same group of mice). (**G**) Digital photographs of tumors. Results are presented as mean ± SD. ** $p \leq 0.01$, * $p \leq 0.05$, ns, not significant.

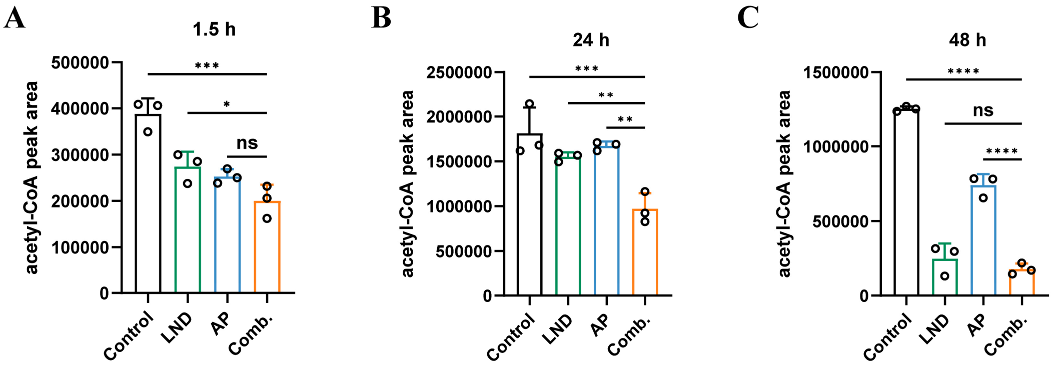

Figure S4. Effect of lonidamine, apigenin, and combination treatment on acetyl-CoA levels in MC38 Cells. (**A**–**C**) acetyl-CoA levels in MC38 cells (treated with 140 μM LND, 14 μM AP, or a combination of both) were measured using UPLC-MS/MS at 1.5 h (**A**), 24 h (**B**) and 48 h (**C**). Results are presented as mean ± SD. *** $p \leq 0.001$, ** $p \leq 0.01$, * $p \leq 0.05$, ns, not significant.
